# Breast cancer protection by genomic imprinting in close kin families

**DOI:** 10.1186/s12881-017-0498-0

**Published:** 2017-11-21

**Authors:** Srdjan Denic, Mukesh M. Agarwal

**Affiliations:** 1 Department of Medicine, College of Medicine and Health Sciences, United Arab Emirates University, PO Box 17666, Al Ain, Abu Dhabi UAE; 2 Department of Pathology, California University of Science and Medicine, 217 E Club Center Drive, San Bernardino, CA 92408 USA

**Keywords:** Public health, Mate selection, Epigenetics, Homozygosis, Heterosis, Arabs, Pakistan

## Abstract

Human inbreeding generally reduces breast cancer risk (BCR). When the parents are biologically related, their infants have a lower birth weight due to smaller body organs. The undersized breasts, because of fewer mammary stem cells, have a lower likelihood of malignant conversion. Fetal growth is regulated by genomically imprinted genes which are in conflict; they promote growth when derived from the father and suppress growth when derived from the mother. The kinship theory explicates that the intensity of conflict between these genes affects growth and therefore the size of the newborn. In descendants of closely related parents, this gene clash is less resulting in a smaller infant. In this review, we elucidate the different mechanisms by which human inbreeding affects BCR, and why this risk is dissimilar in different inbred populations.

## Background

The link between human consanguinity and malignancy is important for understanding carcinogenesis. Cancer has a strong genetic component and human consanguinity, still very common in many parts of the world, increases gene homozygosis. Homozygosis of low penetrance tumor genes, and its frequency in the population, can increase or decrease the risk of cancer [1–6]. Homozygosis of mutated tumor suppressor genes in stem cells with double dose can cause either early abortion or early childhood cancer [7–9]. Consanguinity can lower breast cancer risk (BCR) as homozygosis of mutated DNA-repair genes like *BRCA1* and *BRCA2*, being incompatible with life, are not transmitted to the next generation [10–12]. Homozygotes of abnormal mismatch repair genes (e.g., *MLH1, MSH2, MSH6*) that cause a severe cancer syndrome, also fail to reproduce [13]. Thus, inbreeding should clear the consanguineous population of these tumor genes and inherited cancer syndromes should be less common in consanguineous (compared to non-consanguineous) populations. However, multiple other factors are involved: parents with a low cancer risk can also produce offspring with a higher cancer risk by negative heterosis, i.e., offspring exhibit more negative qualities than their parents [14–16]. Thus, the cumulative (and often competing) outcome of different consanguinity-cancer models is difficult to predict due to varying frequency of the different cancer genes and varying environmental aspects involved in carcinogenesis in different populations. As a result, both an increase and a decrease in risk of different cancers has been reported in different consanguineous populations [17–27]. Similarly, inconclusive links have been reported between cancer risk and autozygosity (measured by size of the regions of homozygosis in human genome) in different world populations [28–34].

The various studies on BCR in inbreeding families, similar to other types of cancer, have also produced contradictory results. BCR is lower in Arab inbreeding families than non-inbreeding families [18–23]. However, the BCR is increased in inbreeding families from Pakistan [24–27]. In this review, we deliberate how human consanguinity can lower BCR through genomic imprinting and why some other mechanisms of inbreeding may increase it.

## Human consanguinity: Prevalence, types of marriages and measures of relatedness

**Fig. 1:**
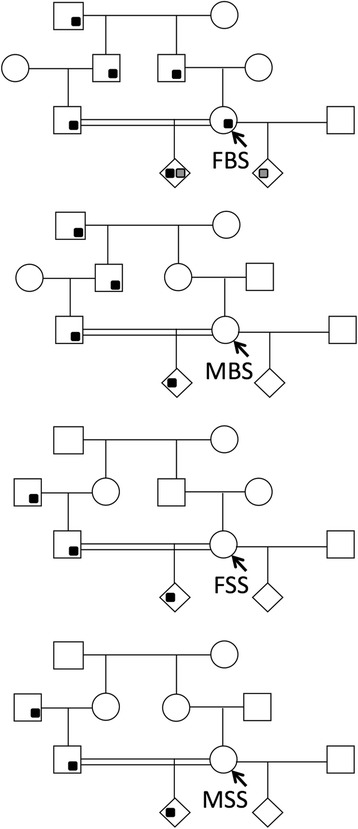
Inheritance of paternally-derived genomically-imprinted gene in four types of first cousin families defined by the cousin wife marries: FBS, father’s brother’s son; MBS, mother’s brother’s son; FSS, father’s sister’s son; MSS, mother’s sister’s son. Only in FBS union paternally-derived and genomically-imprinted gene (black dot) continuously retains all three identities (DNA sequence, biochemical structure and functionality) over three generations; in the fetus, one of its copies becomes maternally derived (gray dot)

Human consanguinity is more frequent than generally appreciated. Worldwide, 1.1 billion of people have consanguineous parents [35]. Such unions are most common in the developing societies of North Africa, the Middle East and South Asia. In these populations, 10% to 50% of all marriages are between close kin: first cousins, double first cousins, first cousins once removed and second cousins. Since the latter unions do not increase biological risks of inbreeding, they are not considered consanguineous. Half of all consanguineous marriages are between first cousins; a woman can be married to only one of her four first cousins: father’s brother’s son (FBS), father’s sister’s son, mother’s brother’s son and mother’s sister’s son (Fig. 1). The Arab world comprises of 22 nations of the Middle East and North Africa in which people speak the same language (Arabic) and share the same religion (Islam); however, their genetic heritage is much less uniform. Arab families, in contrast to non-Arab families, arrange FBS unions half the time instead of the expected one fourth. The preference for FBS marriage is a cultural trait universal among Arabs which mandates that every woman’s marriage should be approved by her paternal uncle [36, 37]. Similarly, in double first cousin unions, there are two possible types of cousins and, in first cousin once removed unions there are eight different types of cousins (Table 1). In contrast to first cousin unions, the frequencies of different cousin choices in later two types of unions are unknown. The biological closeness of spouses is defined by the coefficient of relatedness (*R*) which indicates the fraction of autosomal genes two individuals share by common descent (Table 1). The coefficient of inbreeding (*F*) is a fraction of autosomal genes that are homozygous by common descent and measures the risk of genetic harm in offspring of biologically related individuals (*F* = *R* / 2). In any population, the mean *R* correlates with the consanguinity rate, but former is a more precise an indicator of biological closeness.

**Table 1:** Coefficients of relatedness by common descent of autosomal genes (*R*) and genomically imprinted genes inherited from father (*R*_*p*_) and mother (*R*_*m*_) in non-kin and close-kin families

Marriage	Women units with	*R*	*R*_*p*_	*R*_*m*_
Random	Non-kin			
Double first cousin	Father’s brother’s and mother’s sister’s son	0.25	0.25	0.25
	Father’s sister’s and mother’s brother’s son	0.25		
First cousin	Father’s brother’s son	0.125	0.125	
	Father’s sister’s son	0.125		
	Mother’s brother’s son	0.125		
	Mother’s sister’s son	0.125		0.125
First cousin once removed	Father’s paternal uncle’s son	0.0625	0.0625	
	Father’s paternal aunt’s son	0.0625		
	Father’s maternal uncle’s son	0.0625		
	Father’s maternal aunt’s son	0.0625		
	Mother’s paternal uncle’s son	0.0625		
	Mother’s paternal aunt’s son	0.0625		
	Mother’s maternal uncle’s son	0.0625		
	Mother’s maternal aunt’s son	0.0625		0.0625

## Parental consanguinity and breast cancer

**Fig. 2:**
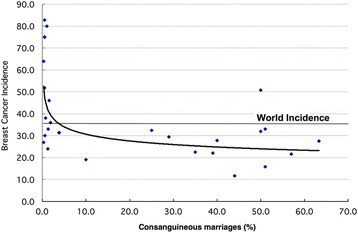
Age-standardized incidence of breast cancer and rate of consanguinity reported from 28 countries. The outlier (incidence 50, consanguinity 50) country is Pakistan. Adapted from reference [12]

The age-standardized incidence of breast cancer is inversely related to the rate of consanguinity (Fig. 2). In five studies from Arab countries, parental consanguinity was protective against breast cancer. In two studies involving the native population of United Arab Emirates, a) BCR in women born to consanguineous parents was half (50%) compared to women of non-consanguineous parents; this protective effect was greater among women less than 50 years old (*p* = 0.02) [18], and b) breast cancer patients had a lower mean coefficient of inbreeding (*p* = 0.19) and their parents were less often first and double first cousins (*p* = 0.09) than the parents of non-cancer controls [19]. In another case-controlled study from Qatar, breast cancer patients had a lower mean coefficient of inbreeding than controls (*p* = 0.0125) [21]. In Morocco, both consanguinity rate and mean coefficient of inbreeding were lower among breast cancer patients than matched controls [22]. Similarly, a statistically significant protective effect of parental consanguinity against breast cancer was reported from Tunisia [23]. One possible explanation for this observation is the protective effect of homozygosis of some low penetrance breast cancer gene(s) common in all Arab populations. Indeed, in a study from Tunisia, homozygote of one variant of the *P53* gene was associated with a lower BCR [38]. However, the same genotype in the Saudi population was reported to increase the BCR [39]. In Arab populations only a few specific and more of commonly shared variants of breast cancer susceptibility genes have been identified [40]. In theory, another possibility is that consanguinity protects against breast cancer by decreasing the number of *BRCA1* and *BRCA2* cancer cases as homozygotes of these genes are early aborted [10–12]. In Arabs, these germline mutations are few and those specific for the population have not unequivocally proven to be carcinogenic [41, 42]; hence, the breast cancer protection by parental consanguinity is largely unexplained.

In contrast to the decrease in risk, breast cancer in Pakistan positively correlates with parental consanguinity [24–27]. Pakistan is a country with an unusually high incidence of breast cancer (outlier in Fig. 2); among its nationals, the incidence of breast cancer is significantly higher compared to its neighboring countries [43]. A high frequency of *BRCA1* and *BRCA2* mutations has been proposed to explain these epidemiological findings. However, most of the gene variants are unique to Pakistan, and they have not been convincingly shown to be carcinogenic [24–26]. In addition, consanguinity should protect against breast cancer caused by *BRCA1* and *BRCA2* which is not a case in this population. In the absence of any obvious environmental risk factor for breast cancer that contrasts Pakistan from its neighbors, a higher frequency of low penetrance cancer genes that increase cancer risk in homozygotes is a more likely explanation. Until now, however, only a few of the known low penetrance breast cancer genes have been studied and none of them can explain the high breast cancer rates in both general population and daughters of consanguineous parents [2, 26]. In this population, however, new mutations of *CHEK2* gene, which moderately increases risk of breast cancer in heterozygotes, were found in rare patients from a few tested ethnic groups [44, 45]. The homozygotes of *CHEK2* mutation are at higher risk of breast cancer development than heterozygote which could explain increased BCR in both general population and daughters of consanguineous parents [46]. Nonetheless, a very low frequency of this gene cannot explain high rate of breast cancer in Pakistan.

Another possible mechanism that could increase breast cancer rate among Pakistani women is negative heterosis (NH) [47]. This phenomenon of NH was noticed almost a century ago in studies of crossing and back crossing of different inbred subspecies of animals which produced offspring with a higher cancer rates than in both parents [14–16]. NH, in contrast to positive heterosis (PH) (hybrid vigor), signifies that the offspring have more negative qualities than their parents. Recently, it has been shown that heterosis is the result of disrupted genomic imprinting in the offspring of two subspecies of inbred parents [48]. In humans, possible examples of PH are bigger daughters from mixed marriages (as seen in one tribal region of India), and NH has been proposed as a cause of the exceptionally high incidence of breast cancer in Pakistan [47, 49]. The hypothesis is based on the fact that after a long history of inbreeding in numerous kinship groups (tribes, biradaris, castes) in the region, there was a sudden and violent migration of unprecedented proportions. In 1947, when the Republic of Pakistan was formed, members of inbred groups were forcefully mixed producing inter-group unions that, like NH in animal studies, could contribute to the extra breast cancer in this population. In short, despite the many hypotheses, the reasons for the higher rates of breast cancer in Pakistan, overall and in consanguineous families, remain largely undetermined.

## Parental consanguinity and newborn size

The lower newborn weight in consanguinity is a less well known phenomenon that remains largely unexplained. In a large (*n* = 10,289) and well-controlled study, infants of consanguineous parents were 1.8% smaller (than newborns of non-consanguineous parents) [50]. In this same study, a review of 11 previous studies showed that in ten of them the babies of consanguineous parents were, on an average, 20 to 221 g smaller. This difference was statistically significant in four studies; in the remaining studies, where the results did not achieve statistical significance, the nutritional deprivation was pronounced, the sample size was small and covariates of fetal growth were not controlled. In similar studies from Turkey, inbred newborns were not smaller but inbred school-aged-males (females were not studied) were significantly smaller than non-inbred controls [51, 52]. In all these consanguinity studies, newborns with congenital disorders were excluded from the analysis.

## The importance of newborn size

In general, a newborn’s size at birth (i.e., the weight, the length and the head circumference) is determined by both genetic and environmental factors [53]. In one study, causes of variation in birth size was estimated to be as follows: fetal genes, 30%; maternal genes, 20%; environmental factors, 15%; and unknown factors, 35% [54]. The mother’s height, weight, and weight at birth are stronger determinants of the baby size than the same parameters of the father. Also, many environmental factors are involved like birth order and, during pregnancy, mother’s illnesses, caloric intake and use of alcohol, tobacco and hormones [54, 55]. Any study addressing a potentially new cause affecting fetal growth must account for the effects of multiple independent cofounders determining newborn size.

From an evolutionary perspective, bigger individuals survive better than smaller ones, and the birth size correlates with body size during adulthood [54]. From a medical perspective, smaller birth size carries an increased risk of chronic nutritional disorders (obesity, diabetes mellitus type 2, fatty liver, hypertension, cardiovascular diseases) and, in women, a decreased risk of premenopausal breast cancer [55, 56].

## Birth size and breast cancer

The neonatal birth size is a well-established risk factor for breast cancer. Studies during the last two decades, from several countries, have consistently shown that birth weight and birth length positively correlate with breast cancer in women <50 years old [57–63]. Bigger babies have larger mammary glands and the number of mammary stem cells is proportional to its size [64–66]. A denser shadow of the breast radiogram is associated with a higher mammary gland mass and a higher BCR [67]; furthermore, bigger babies have a higher number of circulating hematopoietic stem cells [68]. The newborn size and the number of stem cells positively correlate with blood levels of several growth promoting hormones and the expression of at least two genomically imprinted genes that affect fetal growth: positively with insulin-like growth factor 2 (*IGF-2*) (which is expressed when inherited from father and inactivated when inherited from mother), and negatively with *PHLDA2* (which is expressed when inherited from mother and genomically imprinted when inherited from father) [69–75]. In short, studies have established a plausible link between the expression of genomically imprinted genes and the risk of malignant transformation in the breast.

## Genomically imprinted genes and genetic conflict theory

The change in gene expression (without the change in DNA nucleotide sequence) is produced by methylation, histone modification, and small RNA interference (imprinting). Generally, with few exceptions, imprinting inactivates genes. Genomic imprinting is a special form of gene inactivation depending on parent from whom it originates. As a rule, a gene in the fetus if inherited from mother is suppressed while the same gene if inherited from father is expressed. This results in mono-allelic inheritance and, in effect, transforms one into two genes, each with identical DNA nucleotide sequence but with different biochemical structure (e.g., with methyl groups or without methyl groups) and function (decreased or increased expression) depending on parent of origin. In humans, genomic imprinting is confined to about 100 genes that regulate cell proliferation during fetal and early postnatal development, behavior and cognition, and are expressed mostly in the placenta and the brain [76, 77]. The evolution of genomic imprinting is explained by the kinship theory [78–81].

### Kinship (genetic conflict) theory of genomic imprinting

The theory of genomic imprinting (also called genetic conflict theory) posits that, in mammals, the genomic imprinting of genes is a consequence of the interactions between the fetus, the mother, the father and their genes, as follows: a) both maternal- and paternal-derived developmental genes regulate growth of the fetus; b) the mother provides disproportionally more resources for the offspring’s growth than the father; c) mothers can have offspring from more than one male (polyandry). Bigger babies survive better but they also utilize more resources from the mother, which could lower her fitness by decreasing her resources available for future offspring. To conserve her resources and maximize fitness, mother’s growth promoting genes are suppressed. On other hand, the father’s genes are expressed to promote growth as this increases the odds of their survival due to a bigger baby. In short, the maternally-derived and paternally-derived genes in the fetus are in conflict about how much growth to promote as this determines the number of their copies in future generations due to a) asymmetry of biological investments in their progeny and b) polyandry which creates different future life trajectories of their gene copies [78–81]. In other words, genomic imprinting emerged during evolution as a mechanism which increases the inclusive fitness of genes. Thus, anything that would affect polyandry is expected to affect the intensity of conflict between genomically imprinted genes and size of the fetus. In one animal study, female rats mated with three males produced bigger pups than females mated with one male [82]. Likewise, in humans, anything that increases inclusive fitness is expected to decrease the intensity of gene conflict and produce smaller babies.

## Genetic conflict is lower in consanguineous families

In a first cousin marriage, the wife carries 0.125 of autosomal genes of her husband by common descent. In future, if she conceives a child with another man, that child would carry 0.0625 of the genes of her current husband, her first cousin and the child’s uncle. In her current child, half of those 0.0625 genes are identical by common descent (Fig. 1). In contrast, in a non-cousin (random mate) marriage, none of the genes of current father will be in the other man’s child. In close-kin families, therefore, polyandry produces less conflict between parental genes in the fetus. The expected conflict reduction is proportional to *R* of parents and is shown in Table 1. The kinship theory of genomic imprinting supports the findings of smaller babies in consanguineous families [50]. This theory also predicts that babies of double first cousins will be smaller than babies of first cousins, which in turn are expected to be smaller than babies of first cousins once removed.

However, the genomically imprinted genes are more important for fetal growth than other genes. As per the kinship theory, they are genomically imprinted for that very reason and, consequently, they exert a stronger effect on size of newborns in any consanguineous family. The coefficient of relatedness of the genomically imprinted paternally-derived (*R*_*p*_) and maternally-derived genes (*R*_*m*_), however, is different from the non-genomically imprinted genes (*R*) and are shown in Table 1. Among 14 different kinds of consanguineous families, only in three *R*_*p*_ > 0; in such families, the production of smaller babies is expected. Among four types of first cousin families, likewise, only the FBS family type (more common amongst Arabs) is expected to produce significantly smaller newborns.

## Breast cancer protection by parental consanguinity: Summary and conclusion

Epidemiological observations have helped to uncover many novel mechanisms of carcinogenesis [83]. Several studies suggest that human inbreeding protects against breast cancer. In general, consanguineous parents produce smaller newborns and smaller newborns develop breast cancer less frequently later in life. The reason for smaller infants in close-kin families is elucidated by the kinship theory of genomic imprinting. As per this theory, growth promoting genes inherited from father and mother are in conflict; the intensity of this conflict controls fetal growth and, ultimately, the size of newborn. In the offspring of close-kin marriages, the intensity of genetic conflict is less which results in smaller newborns.

In Arab women, the decrease in BCR by parental consanguinity could be a result of i) decreased gene conflict that is enhanced by a larger number of first cousin marriages of the FBS type, ii) protective effect of homozygosis of some still unidentified breast cancer gene(s), or iii) additive protective effect of both these mechanisms against breast cancer. However, in Pakistan parental consanguinity increases BCR**.** As explained earlier, BCR reduction by decrease gene conflict may be exceeded by increased risk due to the following factors: i) cancer increase due to NH, ii) homozygosis of unidentified breast cancer susceptibility genes or iii) the additive effect of both these mechanisms.

There are several cofounders in consanguinity – cancer risk besides infant size. Theoretically, the frequency of lethal cancer alleles is reduced by inbreeding in proportion to its duration and intensity. Inbreeding coexists with socioeconomic underdevelopment, which is associated with lower risk factors for breast cancer (e.g., earlier pregnancies, longer nursing, decreased use of alcohol and tobacco). In consanguineous populations, women generally marry earlier and earlier first pregnancies protects against breast cancer [36]. In some (but not all) studies, the daughters of consanguineous parents marry consanguineously more often, and they could be better protected by an earlier first pregnancy [18, 84]. Polyandry, a theoretical determinant of baby size, varies between societies. Finally, a fathers’ investment in offspring, another determinant of baby size, is a cultural trait that varies between societies, and it is a possible cofounder in BCR.

In future studies, for more clarity, the effect of autosomal gene conflict on birth size can be verified by comparing the parental coefficient of relatedness, the offspring coefficient of inbreeding and infant size from consanguineous and non-consanguineous families in the same population. The validity of genomic imprinting hypothesis of inbreeding can be tested by comparing the parental coefficient of relatedness of genomically imprinted genes and birth size. Furthermore, measuring growth hormones like IGF-2 in fetal blood and expression of developmental genes in the placenta (from women married to different types of cousins) would provide additional pieces of the puzzle. Then, we can be sure of the relative role of genomic imprinting in reducing the risk of breast cancer in inbred populations – and add further to our understanding of carcinogenesis.

## Abbreviations

*F*: Coefficient of inbreeding
FBS: Father’s brother’s son
FSS: Father’s sister’s son
IGF-1: Insulin-like growth factor 1
MBS: Mother’s brother’s son
MENA: Middle East and North Africa
MSS: Mother’s sister’s son
*R*: Coefficient of relatedness
*R*_m_: Coefficient of relatedness of maternally derived gnomically imprinted genes
*R*_p_: Coefficient of relatedness of paternally derived gnomically imprinted genes
UAE: United Arab Emirates
